# FORECASTING THE REGIONAL DISTRIBUTION OF HOME CARE PATIENTS USING BIG DATA OF INSURANCE CLAIMS IN JAPAN: 2015 TO 2045

**DOI:** 10.1093/geroni/igac059.557

**Published:** 2022-12-20

**Authors:** Yasuhiro Nakanishi, Yuichi Nishioka, Yukio Tsugihashi, Tomohiro Kakinuma, Tatsuya Noda, Tomoaki Imamura, Manabu Akahane

**Affiliations:** National Institute of Public Health, Wako, Saitama, Japan; Nara Medical University, Kashihara, Nara, Japan; Nara Medical University, Kashihara, Nara, Japan; National Institute of Public Health, Wako, Saitama, Japan; Nara Medical University, Kashihara, Nara, Japan; Nara Medical University, Kashihara, Nara, Japan; National Institute of Public Health, Wako, Saitama, Japan

## Abstract

Regional distribution of home care patients and future demand in Japan are unknown. This study aimed to reveal the actual situation of home care patients by region and forecast demand up to 2045. Linked complete health and long-term care insurance claims data on Nara Prefecture (around 1% of the total population and area of Japan) patients aged 75 years or older who received planned and/or urgent medical treatment by physician home visit between April 2015 and March 2016 were extracted and analyzed by sex, age group, and municipality. We calculated the proportion of home medical care utilization and projected the number of home care patients for every five-year period up to 2045 across five administrative areas of the medical service in Nara Prefecture. Data on 12,656 patients, including 1,455 aged 75–79 years, 2,753 aged 80–84, 3,854 aged 85–89, and 4,594 aged 90 years or older, were extracted. The current proportion of patients receiving home medical care (unadjusted for age) by medical service administrative area showed a difference of up to 1.6 times for those aged 90 years or older. Results of forecasting showed a marked increase in the number of patients aged 90 years or older, with overall numbers continuing to increase up to 2040, reaching a maximum of around 25,759 then decreasing thereafter. The future increase in home care patient numbers could vary by age and area, and our findings suggest that public health policy based on the future demand in each area will be required.

